# Thermal Adaptation in *Liriomyza trifolii* (Diptera: Agromyzidae): From Interspecific Competition to Mechanisms

**DOI:** 10.3390/insects16090957

**Published:** 2025-09-11

**Authors:** Ya-Wen Chang, Jing-Ya Zhao, Yu-Cheng Wang, Yu-Zhou Du

**Affiliations:** 1 Institute of Applied Entomology, College of Plant Protection, Yangzhou University, Yangzhou 225009, China; changyawen@yzu.edu.cn (Y.-W.C.);; 2 Jiangsu Province Engineering Research Center of Green Pesticides, Yangzhou University, Yangzhou 225009, China; 3 Department of Entomology, University of Minnesota, St. Paul, MN 55108, USA

**Keywords:** *Liriomyza trifolii*, interspecific competition, thermal tolerance, rapid cold hardening, heat shock proteins

## Abstract

Global climate change has intensified temperature fluctuations, significantly impacting insect populations. Thermal tolerance has emerged as a critical determinant of species distribution and invasion potential. *Liriomyza trifolii*, an economically important invasive pest, has been rapidly expanding in southeastern coastal regions of China, gradually displacing its congeners *L. sativae* and *L. huidobrensis*. This competitive advantage is closely associated with its superior thermal adaptation strategies. Here, we first examine the temperature-mediated competitive dominance of *L. trifolii*, then systematically elucidate the physiological, biochemical, and molecular mechanisms underlying its temperature tolerance, revealing its survival strategies under extreme temperatures. Notably, *L. trifolii* exhibits a lower developmental threshold temperature and higher thermal constant, extending its damage period, while its significantly lower supercooling point confers exceptional overwintering capacity. Physiologically, rapid cold hardening (RCH) enhances cold tolerance through glycerol accumulation and increased fatty acid unsaturation, while heat acclimation improves thermotolerance via a trade-off between developmental processes and reproductive investment. Molecular analyses demonstrate that *L. trifolii* combines the low-temperature inducible characteristics of *L. huidobrensis* with the high-temperature responsive advantages of *L. sativae* in heat shock protein (Hsp) expression patterns. Transcriptomic studies further identify differential expressions of lipid metabolism and chaperone-related genes as key to thermal adaptation. Current research limitations include incomplete understanding of non-Hsp gene regulatory networks and laboratory–field adaptation discrepancies. Future studies should integrate multi-omics approaches with ecological modeling to predict *L. trifolii*’s expansion under climate change scenarios and develop temperature-based green control strategies.

## Simple Summary

Global climate change has intensified temperature variability, affecting insect populations and making thermal tolerance a key factor in species distribution and invasion success. The invasive pest *Liriomyza trifolii* has expanded rapidly in southeastern China, outcompeting its relatives *L. sativae* and *L. huidobrensis*, largely due to superior thermal adaptation. This paper explores *L. trifolii*’s temperature-mediated dominance, uncovering physiological, biochemical, and molecular mechanisms behind its resilience. Key adaptations include a low developmental threshold temperature, high thermal constant (extending its damage period), and a low supercooling point (enhancing overwintering). Physiologically, cold hardening increases glycerol and fatty acid unsaturation, while heat acclimation involves trade-offs between development and reproduction. Molecularly, *L. trifolii* combines advantageous heat shock protein (Hsp) expression patterns from both congeners, and transcriptomics highlight lipid metabolism and chaperone genes as critical for thermal adaptation. Current gaps include incomplete gene regulatory network knowledge and lab-field discrepancies. Future research should combine multi-omics and ecological modeling to predict *L. trifolii*’s spread under climate change and develop temperature-based pest control strategies.

## 1. Introduction

Global climate change has significantly altered temperature regimes, posing substantial threats to biodiversity and profoundly impacting the distribution and abundance of natural populations [1]. As ectothermic organisms, insects are particularly vulnerable to environmental temperature fluctuations, with thermal tolerance being a critical determinant of their survival and persistence in specific ecological niches [2,3]. Temperature directly affects the geographical distribution and activity patterns of insects, thus playing a key role in their invasion potential [4,5]. The ability to adapt to thermal changes through behavioral avoidance or physiological domestication is a key factor in determining a species’ ability to establish, reproduce, and ultimately achieve successful invasion and colonization. Through a long evolutionary process, insects have developed a variety of strategies to cope with extreme temperature conditions [6,7].

The leafminer flies (*Liriomyza* spp.) are a globally significant pest that affects vegetables and ornamental crops and has become a major invasive species in China’s agricultural system [8,9,10]. Both the larval and adult stages can cause severe damage to crops; in larval form they create serpentine mines in the leaves, damaging photosynthesis, while the feeding and egg-laying wounds of adults provide entry points for pathogenic microorganisms [8,10]. Among the numerous *Liriomyza* species that exhibit rapid interspecific competition and substitution, three polyphagous invaders, *L. trifolii* (Burgess), *L. sativae* Blanchard, and *L. huidobrensis* (Blanchard), have caused particularly serious harm to China [10]. Originating in the Americas, *L. trifolii* was first detected in Guangdong Province in 2005 and has since spread rapidly, primarily causing outbreaks in southeastern coastal regions [11]. This species demonstrates the competitive displacement of its closely related species, showing a clear trend toward becoming the dominant species [12,13]. Multiple factors influence this competitive replacement among invasive *Liriomyza* congeners, including reproductive interference, insecticide resistance, host plant preferences, natural enemies, and particularly temperature adaptation [14,15,16]. Notably, temperature emerges as the most critical factor governing their distribution patterns and competitive outcomes [17,18].

This review systematically examines: (1) the temperature-mediated competitive advantages of *L. trifolii*; (2) the underlying physiological, biochemical, and molecular mechanisms of its thermal tolerance; and (3) current research limitations and future directions in this field. By integrating recent findings, we aim to provide a comprehensive understanding of how thermal adaptation contributes to the invasion success of this economically important insect pest, as well as to assist in developing temperature-mediated field control strategies.

## 2. Thermal Adaptation Advantage Serves as a Key Competitive Determinant in *L. trifolii*

Temperature serves as a fundamental regulator of insect population dynamics, profoundly influencing growth, development, reproduction, and behavioral activities. When competing closely related species coexist, and thermal maladaptation in one species leads to reduced survival, feeding efficiency, adult flight capacity, and oviposition performance, ultimately resulting in competitive displacement [19,20]. Notably, thermal tolerance, especially cold resistance, represents a key life-history trait that affects the completion of the life cycle, successful overwintering, and habitat expansion of invasive *Liriomyza* species [21].

Comparative studies of three important congeneric *Liriomyza* species (*L. sativae*, *L. trifolii*, and *L. huidobrensis*), reveal distinct thermal adaptation strategies that shape their ecological competitiveness under different temperature conditions. An indoor competitive assay was conducted between two *Liriomyza* species, initiated with equal population densities, under four temperature conditions (14 °C, 21 °C, 28 °C, and 35 °C). At their respective optimal developmental temperatures, *L. sativae* and *L. trifolii* exhibit similar reproductive and competitive abilities. However, under stress temperature and pressure conditions, including high (35 °C) and low (14 °C) temperatures, *L. trifolii* demonstrated greater adaptability, with a smaller decline in fecundity than that of *L. sativae.* This indicates that it has enhanced thermal tolerance and competitive advantages at suboptimal temperatures [12]. In contrast, the reproductive response of *L. huidobrensis* within the range of 18–30 °C shows a unimodal pattern, and its competitiveness decreases with the increase in temperature. The reproductive output and competitive ability of *L. sativae* both exhibit characteristics positively correlated with temperature, which leads to their thermal niche differentiation: *L. huidobrensis* dominates in cooler environments, whereas *L. sativae* is more likely to survive in warmer conditions [22,23]. Further comparative analyses of *L. trifolii* and *L. sativae* have revealed their subtle adaptive strategies. Although *L. sativae* maintains a relatively high overall reproductive capacity over a wide temperature gradient (20–33 °C), *L. trifolii* matures at a faster rate. The temperature-dependent survival advantage is quite obvious: *L. sativae* perform better at low temperatures (20 and 25 °C), while *L. trifolii* have a higher adaptability at high temperatures (31 and 33 °C). Interspecific competition was quantified by comparing the net reproductive rates (R_0_) of single species and mixed populations under four isothermal conditions (20, 25, 31, and 33 °C). Both species showed a significant decrease in R_0_ in the mixed population, confirming the intense competition between them. It is worth noting that the competitive level shifts with temperature: *L. sativae* has an advantage at lower temperatures, while *L. trifolii* gains a competitive edge under high-temperature conditions [16]. These findings provide key insights into the ecological distribution of these economically significant leaf miners and the thermal adaptation mechanisms behind their successful invasion. Temperature-mediated competitive asymmetry may explain the differences in their colonization and diffusion in different geographical regions, which provides a reference for prediction models of pest management and climate-driven distribution range expansion.

Among the three closely related *Liriomyza* species, the developmental threshold temperature of each developmental stage of *L. trifolii* is lower than that of *L. sativae*. This indicates that, under field conditions, the theoretical emergence time of the overwintering generation of *L. trifolii* should be earlier than that of *L. sativae* [24,25,26]. However, *L. trifolii* requires a higher effective accumulated temperature to complete its generation development, which means that its annual crop damage period may be longer than that of *L. sativae* [27,28]. In contrast, *L. huidobrensis*, which is adapted to high-latitude and high-altitude environments, has the lowest values for both developmental threshold and effective accumulated temperature among the three species [29] (Table 1). Physiological studies reveal striking differences in cold resistance mechanisms. The supercooling points (SCPs) of *L. trifolii* and *L. huidobrensis* are significantly lower than that of *L. sativae*, indicating superior freeze-avoidance adaptations [30,31,32] (Table 1). Consistent with these findings, comparative overwintering experiments have confirmed that the that *L. trifolii* can survive at higher altitudes, and its cold resistance is significantly better than that of *L. sativae*, with a higher survival rate in extremely low temperatures. The exceptionally low SCP of *L. trifolii* indicates that it may successfully overwinter in most climate zones in China.

Due to the highly similar morphological characteristics and ecological habits of *L. sativae* and *L. trifolii*, researchers have been prompted to conduct more in-depth comparative studies on their temperature adaptability biology [33]. Research shows that *L. trifolii* demonstrates multi-dimensional advantages in temperature adaptability: it not only maintains a competitive edge in high-temperature environments [16], but its significantly lower SCP also demonstrates outstanding cold resistance [32]. Compared with its closely related species, *L. trifolii* has a lower initial developmental temperature, a higher effective accumulated temperature requirement, and prominent cold resistance. These adaptive characteristics jointly contribute to its earlier seasonal emergence time and longer annual activity cycle [26]. The synergistic effects of these physiological adaptation characteristics give *L. trifolii* a competitive advantage over a wide range of temperatures. The ability of this species to maintain its population survival under both extreme conditions of high and low temperatures demonstrates its significant phenotypic plasticity, which may explain the reason for its successful colonization in different agricultural ecosystems. These findings highlight the critical need for further exploration of the physiological, biochemical and molecular mechanisms of its temperature tolerance. Such research can not only provide important basis for formulating climate-adaptive pest control strategies, but also be used to predict the expansion trend of the distribution area of this species under the current background of climate change.

## 3. Physiological and Biochemical Mechanisms of Thermal Tolerance in *L. trifolii*

The rapid enhancement of cold tolerance in insects through brief sublethal hypothermia treatment, a phenomenon known as “rapid cold hardening” (RCH), is a crucial but understudied adaptation mechanism in insect thermal ecology [34,35]. This physiological plasticity enables pests to cope with sudden temperature changes, which has a significant impact on their overwintering success rate and geographical distribution. A comparative study of *L. sativae* and *L. trifolii* has shown significant interspecific differences in basic cold tolerance and RCH capacity between the two, which provides clues to explain the differences in their invasion success rates in different climate zones. Baseline cold tolerance assays demonstrated that *L. trifolii* exhibits greater inherent cold hardiness than *L. sativae*, with the LT_80_ (lethal temperature for 80% mortality) for 5-day-old pupae measured at −11.6 °C and −8.9 °C, respectively. After being acclimated at 0–5 °C for 4 h and then exposed to LT_80_ temperature for 2 h, respectively, both species of leaf miners showed significant RCH responses during the pupal stage, and the survival rate of the acclimated individuals was significantly higher than that of the non-acclimated control group. However, this plasticity was life-stage dependent: while *L. trifolii* adults exhibited clear RCH effects, *L. sativae* adults showed no measurable hardening response [36]. The superior cold tolerance and broader RCH capacity of *L. trifolii* across life stages likely contributed to its competitive dominance in temperate regions [32]. These physiological adaptations enable *L. trifolii* to withstand unpredictable cold snaps and exploit earlier seasonal activity windows, potentially displacing *L. sativae* in cooler climates. The absence of adult RCH in *L. sativae* may limit its overwintering success in regions with high thermal variability, constraining its range expansion relative to *L. trifolii*. The demonstrated RCH capacity in *Liriomyza* species, particularly *L. trifolii*, underscores the limitations of using static thermal thresholds in predictive models. Current degree-day models and overwintering risk assessments may underestimate pest resilience by failing to account for such rapid physiological adjustments. Incorporating RCH dynamics into forecasting frameworks could improve the accuracy of phenological predictions and inform the timing of control measures. Furthermore, these findings highlight the need for season-specific management strategies, particularly in early spring and late autumn when RCH-enhanced survival may facilitate population persistence.

Recent comparative studies on *Liriomyza* species have elucidated distinct physiological and biochemical strategies for RCH [36]. Our extensive investigations into *L. trifolii* have revealed significant ontogenetic variation in RCH capacity, with 7-day-old pupae emerging as the most cold-adaptable developmental stage. Following 4 h hardening at 1 °C, these pupae displayed a remarkable increase in cold tolerance, with survival rates at lethal temperatures jumping from 20% to over 80% during subsequent 2 h exposure. This protective effect showed complete reversibility, disappearing entirely after 4 h of recovery at 26 °C. The underlying mechanisms involved three synergistic adaptations: First, RCH significantly enhanced supercooling capacity across all developmental stages, with SCP reductions ranging from approximately 1 °C in 5–7-day-old pupae to 1.7 °C in adults. Second, we observed comprehensive membrane lipid restructuring characterized by increased fatty acid unsaturation through both elevated unsaturated fatty acids and decreased saturated counterparts. Third, the response included substantial accumulation of cryoprotective compounds, with glucose and trehalose concentrations increasing 2–3-fold in acclimated adults post-stress [37]. Notably, the transience of these physiological adjustments, including the complete reversal of membrane modifications and cryoprotectant levels, precisely corresponded to the temporal dynamics of RCH induction and decay. These synergistic biochemical changes, particularly the dual strategies of optimizing cell membrane fluidity and accumulating osmotic protectants, not only reveal the outstanding ability of *L. trifolii* to rapidly adapt to heat, but also highlight the metabolic cost of this plastic response. The fact that all measurement parameters are completely reversible indicates that this species has achieved the best evolutionary balance between cold resistance protection and energy conservation. In contrast, *L. huidobrensis*, which has stronger cold resistance, demonstrates a more comprehensive adaptation strategy, involving the coordinated regulation of membrane lipid modification and cryoprotectants. This species showed increased palmitic acid content, decreased palmitic acid level, elevated unsaturated/saturated fatty acid ratio, and dynamic changes in various cryoprotectants, and increased trehalose and glycerol content accompanied by decreased glucose level. These findings reveal the evolutionary differentiation of the internal cold domestication mechanism of within *Liriomyza*, where *L. huidobrensis* adopts a multi-dimensional biochemical strategy, integrating membrane fluidity regulation and complex cryoprotectant dynamics, while its closely related species mainly rely on glycerol accumulation [38]. This differentiated adaptation pattern may reflect the specific ecological adaptation of species to different thermal environments. Among them, the more sophisticated cold resistance mechanism of *L. huidobrensis* gives it a more competitive edge in cold habitats. The interspecific variation in the rapid cold domestication mechanism provides important insights for the evolutionary ecological study of insect temperature adaptation and can offer a scientific basis for predicting the response of species distribution to climate change.

Thermal stress adaptation is a critical process for the survival and reproduction of insects in diverse ecological environments. The geographic distribution of a species is partly determined by its ability to adapt to temperature variations. Therefore, comparing differences among geographically distinct populations can provide important insights into the evolutionary mechanisms underlying thermal adaptation [2,3]. A comparative study of different geographical populations of *L. trifolii* has revealed significant geographical differentiation characteristics of its high-temperature adaptability. Multiple heat tolerance indicators show that the performance of the tropical Hainan population has always been better than that of the temperate Jiangsu population: the Hainan strain has a significantly higher critical thermal maximum (Ct_max_), with the survival threshold of adult insects reaching 43 °C before it begins to decline, while that of the Jiangsu population is 42 °C. Although the resistance of the two populations to acute heat stress was comparable, in the chronic heat exposure experiment, the Hainan population demonstrated stronger adaptability—especially after continuous exposure within the range of 42–46 °C for 1 h, where its pupal survival rate was significantly higher [39]. These population differences are highly consistent with the climatic characteristics of their native habitats, indicating that the population in Hainan has adapted to the persistently hot tropical environment there. Although this study evaluated thermal tolerance by comparing the Ct_max_ of two geographically distinct populations, research on *Drosophila* has demonstrated that the male fertility thermal limits are significantly lower than Ct_max_ and serves as a more accurate predictor of both the species’ current distribution and their true thermal tolerance under laboratory conditions. However, similar mechanisms remain unreported in *Liriomyza* species [40]. Laboratory-controlled selection experiments further revealed its adaptation mechanism: moderate thermal domestication can enhance overall fitness by synergistically increasing reproductive output and pupal size [41]. A similar trade-off relationship between temperature and reproduction has also been found in the closely related *L. huidobrensis* [42]. These comprehensive findings indicate that *L. trifolii* has significant adaptive plasticity, and different temperature environments can drive optimized domestication or costly maladaptation, while natural populations maintain a heat tolerance threshold that matches the local environment through the evolutionary process.

## 4. Molecular Mechanisms of Thermal Tolerance in *L. trifolii*

As poikilothermic organisms, insects are significantly influenced by environmental temperature, and their ability to withstand heat stress is a key factor in determining the survival and continuation of a population in a specific ecological niche [6,7]. At present, the research on the molecular mechanisms underlying temperature stress tolerance in leafminer flies still mainly focuses on heat shock proteins (Hsps) [43,44]. Heat shock proteins, as a highly conserved type of stress response proteins that are widely present in living organisms, play a core regulatory role in the growth, development, and survival adaptation of insects [45,46,47]. During stress responses, Hsps primarily function as molecular chaperones by the following: (1) maintaining proper protein folding; (2) facilitating transmembrane protein transport; and (3) preventing precursor protein accumulation [48,49]. Different Hsps interact with target proteins through distinct mechanisms, thereby conferring diverse protective functions. Comparative analysis of temperature adaptation-related gene expression among closely related species provides a quantitative framework for evaluating interspecific differences in thermal stress adaptation.

**Figure 1:**
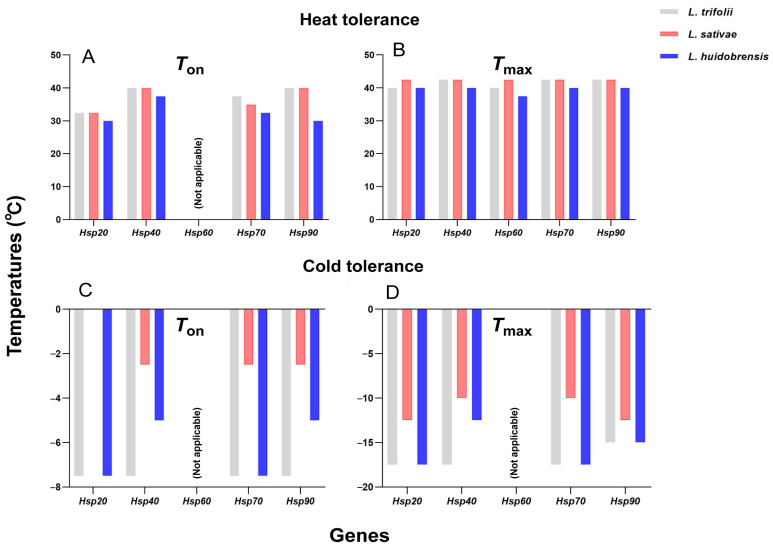
The expression onset (*T*_on_) and peak (*T*_max_) temperatures of Hsps in the three *Liriomyza* species. (**A**) *T*_on_ temperatures under heat stress; (**B**) *T*_max_ temperatures under heat stress; (**C**) *T*_on_ temperatures under cold stress; (**D**) *T*_max_ temperatures under cold stress.

Regarding *Hsp* genes expression in *Liriomyza* species, five different *Hsps* were successfully cloned and characterized from *L. sativae* and *L. huidobrensis*. Their investigations revealed that most *Hsps* were significantly upregulated in pupae exposed to thermal stress, except *Hsp60* which showed no response to cold stress. Notably, both the onset (*T*_on_) and peak (*T*_max_) expression temperatures for *Hsps* in *L. huidobrensis* were generally lower than those in *L. sativae*, suggesting these parameters may explain interspecific differences in thermal tolerance [44]. These findings also highlight the evolutionary trade-offs between thermal stress response, *Hsp* expression, and ecological adaptation, where enhanced thermotolerance in *L. huidobrensis* comes at the cost of reduced fecundity [42]. Building upon previous work, Chang et al. systematically investigated five *Hsp* genes in *L. trifolii*, analyzing their induction patterns under thermal stress and comparing them with orthologous genes in *L. sativae* and *L. huidobrensis* [43]. Their results demonstrated significant differences in *T*_on_ and *T*_max_ values among the three congeneric species. Under cold stress, *L. trifolii* exhibited *T*_on_ and *T*_max_ values 2.5–7.5 °C lower than *L. sativae* but similar to *L. huidobrensis*. On the contrary, under heat stress conditions, these parameters of *L. trifolii* were 2.5–5.0 °C higher than those of *L. huidobrensis*, but comparable to those of *L. sativae* (Figure 1). This unique expression pattern indicates that *L. trifolii* has evolved a heat adaptation strategy that lies between two closely related species, while also possessing the dominant traits of both [43,50]. Further research on two Hsp families (sHsps and Hsp70) that are particularly sensitive to temperature in *L. trifolii* has found the following: (1) these genes were strongly induced under various thermal stress conditions [51,52]; (2) RNA interference studies have confirmed their functional importance [53]; and (3) transcriptional regulation analyses indicated that the expression patterns of these genes [54] were clearly associated with the species’ outstanding thermal adaptability. These findings collectively confirm the core role of Hsps in the temperature tolerance of *Liriomyza* species, and, at the same time, reveal the complex differences in stress response mechanisms among different species.

Comparative transcriptomic analysis of three *Liriomyza* species revealed species-specific gene expression characteristics of significant ecological importance. The research found that the differentially expressed genes (DEGs) of the three species showed significantly different clustering patterns. Among them, the expression profiles of *L. sativae* were highly similar to those of *L. trifolii*, but in sharp contrast to those of *L. huidobrensis.* This difference was particularly reflected in the genes related to key competitive traits such as thermal tolerance, host adaptability, and pesticide resistance. It is worth noting that, compared with *L. huidobrensis*, *L. sativae* and *L. trifolii* have a greater number of upregulated genes, suggesting that their stronger competitive ability may explain their significant ecological advantages [55]. Transcriptomic data provide molecular evidence that *L. sativae* is the most widely distributed leafminer in China, and its extensive distribution may be related to its large number of highly expressed DEGs. Although *L. trifolii* and *L. sativae* share similar differential gene characteristics, their expression patterns exhibit unique adaptive features: under high and low temperature stress conditions, this species significantly enriches genes involved in post-translational modification, protein homeostasis (molecular partner and turnover mechanisms), and lipid transport metabolism [56]. Particularly prominent is that *L. trifolii* specifically regulates the genes of Hsps and epidermal proteins in the response to heat stress. This discovery provides important clues for understanding the molecular mechanism of the species’ competitive advantage. These findings not only demonstrate the value of comparative transcriptomics for understanding invasive species success but also identify key candidate genes that may contribute to *L. trifolii*’s remarkable environmental adaptability and underscore its substantial invasive potential despite currently being less widespread than *L. sativae* in China.

Comparative transcriptomic analyses of RCH mechanisms between *L. sativae* and *L. trifolii* revealed distinct molecular adaptation strategies. *L. sativae* exhibited a more pronounced transcriptional response, showing greater numbers of DEGs in both RCH versus control and RCHCS (RCH + cold stress) versus CS (direct cold stress) comparisons. While both species shared significant enrichment in key functional categories, including fatty acid metabolic enzymes, cuticular proteins, and cytochrome P450 genes, the broader transcriptional reprogramming in *L. sativae* suggests superior RCH capacity that may contribute to its ecological success and widespread distribution across China despite competitive pressure from the more temperature-tolerant *L. trifolii* [57]. After detailed examination of *L. trifolii*’s cold response, the most significantly regulated genes are encoded fat body proteins, cuticular components, and DNA-binding proteins, indicating coordinated physiological adjustments at multiple levels. KEGG pathway analysis revealed extensive metabolic reprogramming, with particular enrichment in energy metabolism pathways (amino acid and carbohydrate processing), fatty acid metabolism, and MAPK signaling cascades. These findings highlight the complex transcriptional regulation underlying *L. trifolii*’s rapid cold tolerance, where integrated modulation of metabolic pathways and stress signaling networks enables effective short-term thermal adaptation [58]. The comparative data suggest that while *L. sativae* may possess greater RCH plasticity through broader gene regulation, *L. trifolii* employ a more targeted metabolic adjustment strategy for cold hardening, reflecting alternative evolutionary solutions to thermal stress in these competing species.

Comparative transcriptomic profiling of *L. trifolii* populations from Jiangsu and Hainan under heat stress revealed fundamentally different molecular adaptation strategies, with the temperate Jiangsu population exhibiting a more extensive transcriptional response compared to its tropical Hainan counterpart. While both populations shared conserved functional enrichment in core stress response pathways, including protein homeostasis (chaperone activity, ER protein processing) and cellular maintenance processes, they diverged markedly in their Hsp expression profiles. The Hainan population demonstrated stronger heat tolerance through the continuous high expression of large-molecular-weight heat shock proteins (*Hsp70*s and *Hsp90*s), while the Jiangsu population relied more on the induced expression of small-molecular-weight heat shock proteins (*sHsp*s and *Hsp40*s) [39,58].

Thermal selection experiments further revealed distinct adaptation strategies, the 35 °C acclimated strain optimized energy distribution by programmed inhibition of Hsps expression during the pupation stage while retaining enhanced stress response capabilities. However, the 40 °C acclimated strain maintains a high level of constitutive Hsps as a first-mover defense mechanism, but incurs significant adaptive costs, including a reduced success rate of emergence [41]. These findings demonstrate that the *L. trifolii* has extraordinary adaptive plasticity. Different populations and thermally domesticated strains have evolved diverse molecular strategies ranging from energy-saving-induced responses to high-cost constitutive defenses, which together form the basis of the pest’s significant survival ability in various temperature environments. The population-specific expression framework of *Hsps*, particularly the tropical population, rely on large-molecular-weight chaperone proteins, while the temperate population rely on sHsps; this provides a molecular-level explanation for the successful colonization of this species in regions with significant climate differences.

## 5. Future Challenges and Opportunities

As ectothermic organisms, the survival of insects is significantly regulated by environmental temperature. This key ecological factor directly determines their survival ability and population continuity in a specific ecosystem [3,59]. The thermal stress tolerance of insects holds significant implications for predicting pest distribution ranges, modeling population dynamics, assessing geographic expansion potential, understanding competitive displacement among closely related species, and developing integrated management strategies, research areas that have gained increasing importance in the context of global climate change [4,5].

When exposed to thermal stress, insects employ various adaptive responses including seeking shelter, modulating cell membrane fluidity, accumulating small molecular cryoprotectants (sugars and alcohols), and upregulating heat shock proteins, etc., [45,60,61]. Adverse thermal conditions primarily affect insect physiology by altering biochemical processes, modifying metabolite profiles, and disrupting gene expression patterns, ultimately impairing growth and development. To counteract these effects, insects have evolved multi-tiered defense mechanisms: behavioral avoidance as the primary response, followed by physiological and molecular adjustments when avoidance is impossible or delayed [62,63]. Beyond heat shock proteins, numerous other thermal tolerance molecular indicators exist, such as genes encoding transient receptor potential (TRP) channels [64], DNA methyltransferases [65], and cuticular proteins [66], and have been identified in various species. However, current research on *L. trifolii* remains relatively limited. This study examines the role of key stress-responsive genes in competitive displacement among leafminer species, yet, beyond heat shock proteins, investigations into other thermal tolerance-related genes remain scarce [21]. Although studies have identified temperature-inducible expression of catalase (CAT) in *L. trifolii*, the induction levels remain relatively low [67]. Similarly, Wang et al. reported temperature-induced upregulation of P450 genes, though their functional significance in thermal adaptation pales in comparison to heat shock proteins [68]. The recent availability of *L. trifolii* genome sequences now enables systematic screening for novel thermal tolerance genes, potentially expanding our understanding beyond the current limited gene repertoire [69].

Future research should prioritize investigating interactive effects between temperature and other factors on thermal tolerance in *L. trifolii*. Wang et al. demonstrated that pesticide pretreatment can modify thermal tolerance [70,71], while studies on its congener *L. huidobrensis* revealed enhanced cold tolerance in *Wolbachia*-infected populations—though similar phenomena remain undetected in *L. trifolii* due to its low natural *Wolbachia* infection rates [72,73]. Notably, the potential influence of host plants on thermal tolerance remains unexplored, despite *L. trifolii*’s endophytic larval development within leaf tissues.

Current research predominantly relies on laboratory studies, raising questions about potential thermal tolerance modifications during long-term rearing. Analogous to findings in *Frankliniella occidentalis* (where constant laboratory conditions enhanced extreme temperature tolerance) [74] and warnings against solely using growth chamber data to predict *L. huidobrensis* invasiveness, similar considerations may apply to *L. trifolii* [75]. The species’ severe summer outbreaks highlight the ecological significance of its robust thermal tolerance. Understanding these adaptive mechanisms could optimize temperature-based control strategies, such as summer greenhouse solarization and winter cover removal. Research on *L. trifolii* thermal tolerance represents an evolving field requiring continued exploration. Future studies should not only deepen our understanding of insect thermal adaptation but also contribute to broader ecological questions regarding insect biodiversity maintenance and climate change impacts on ecosystems, potentially establishing new theoretical frameworks and research paradigms in thermal biology.

## 6. Conclusions

**Figure 2:**
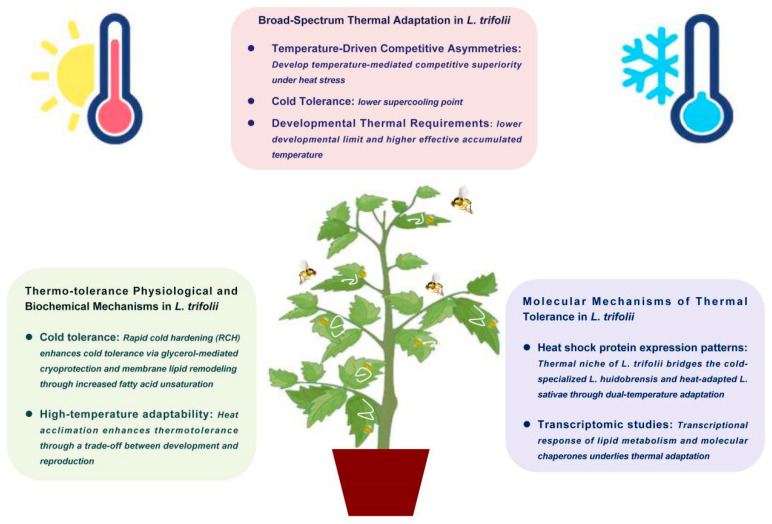
Thermal adaptation strategies in *Liriomyza trifolii*.

The invasive success of *L. trifolii* in agricultural ecosystems is closely tied to its exceptional thermal adaptability, which enables it to outcompete congeneric species across diverse climatic conditions. This review synthesizes current knowledge on the thermal biology of *L. trifolii*, highlighting three key findings: (1) Thermal adaptation as a competitive advantage—*L. trifolii* exhibits superior thermal tolerance compared to *L. sativae* and *L. huidobrensis*, with a lower developmental threshold temperature, enhanced cold resistance (evidenced by a lower SCP), and greater resilience under heat stress. These traits facilitate earlier seasonal emergence, prolonged annual activity, and successful colonization of both temperate and tropical regions. (2) Physiological and biochemical mechanisms—*L. trifolii* has evolved a suite of sophisticated adaptive strategies to thermal stress, primarily through RCH mechanisms. These adaptations include the following: dynamic remodeling of membrane phospholipids to maintain fluidity, strategic accumulation of cryoprotectants (trehalose and glycerol) to prevent ice formation, and rapidly reversible metabolic reprogramming. Comparative studies demonstrate population-specific adaptation patterns, with geographically distinct populations exhibiting divergent thermal responses and lab-adapted strains showing altered tolerance thresholds. Crucially, these thermal adaptations incur fitness costs, manifesting as a fundamental trade-off between enhanced thermal tolerance and reduced reproductive output—a key constraint shaping the species’ evolutionary ecology. (3) Molecular basis of thermal tolerance—transcriptomic analyses reveal that *L. trifolii* employs a multifaceted molecular response to thermal stress, including differential expression of Hsps, cuticular proteins, and metabolic regulators. Its intermediate thermal adaptation strategy—combining traits from both heat- and cold-adapted congeners, which enhances its ecological plasticity and invasive potential (Figure 2). Ultimately, understanding *L. trifolii*’s thermal adaptation not only aids in mitigating its agricultural impact but also provides broader insights into how invasive species respond to global climate change. This knowledge is essential for developing resilient pest management frameworks in an era of shifting environmental conditions.

## Figures and Tables

**Table 1:** Comparison of developmental threshold temperature, effective accumulated temperature, and supercooling point among the three *Liriomyza* species.

Species	Developmental Threshold Temperature (°C)	Effective Accumulated Temperature (°C.day)	Supercooling Point (°C)
*L. trifolii*	8.40 [26]	315.0 [26]	−22.56 [32]
*L. sativae*	9.57 [24]	283.2 [24]	−11.79 [32]
*L. huidobrensis*	7.50 [29]	279.9 [29]	−20.90 [30]

## Data Availability

Data is contained within the article.
